# Biodistribution of ^89^Zr-Radiolabeled Nanoassemblies for Monoclonal Antibody Delivery Revealed through *In Vivo* PET Imaging

**DOI:** 10.1021/acsomega.4c09823

**Published:** 2025-01-28

**Authors:** Ana M. López-Estévez, Amaia Carrascal-Miniño, Dolores Torres, María José Alonso, Rafael T. M. de Rosales, Juan Pellico

**Affiliations:** † Center for Research in Molecular Medicine and Chronic Diseases (CiMUS), Health Research Institute of Santiago de Compostela, University of Santiago de Compostela, 15782 Santiago de Compostela, Spain; ‡ Department of Pharmacology, Pharmacy and Pharmaceutical Technology, School of Pharmacy, University of Santiago de Compostela, 15782 Santiago de Compostela, Spain; § School of Biomedical Engineering & Imaging Sciences, King’s College London, St. Thomas’ Hospital, London SE1 7EH, U.K.

## Abstract

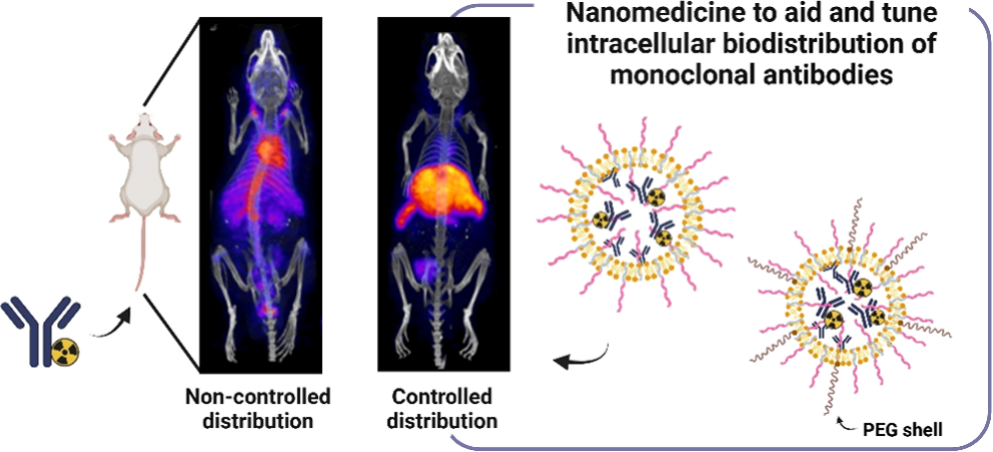

Despite the outstanding performance of monoclonal antibodies (mAbs) in the clinic, their full potential has been hindered due to their inability to cross cell membranes and therefore reach intracellular targets. The use of nanotechnology to deliver mAbs to intracellular domains has been highlighted as a strategy with high potential. Working toward this goal, we have recently developed and validated palmitoyl hyaluronate (HAC16)-based nanoassemblies (HANAs), a novel technology for the intracellular delivery of mAbs in Kirsten Rat Sarcoma Virus (KRAS)-mutated tumors, one of the most prevalent and a challenging intracellular oncoprotein. Despite their success, the pharmacokinetics and biodistribution of these delivery vehicles are still unknown due to their chemical complexity, a challenge common to a large proportion of drug delivery nanomedicines. To support further development and clinical translation, we present an efficient radiolabeling approach with the positron emitter zirconium-89 (^89^Zr) for the *in vivo* evaluation of HANAs by whole-body PET imaging. Additionally, we assessed the impact of PEGylation and size modulation on the biodistribution profile of mAbs using ^89^Zr-radiolabeled PEGylated and non-PEGylated HANAs. Our PET imaging results demonstrated that HANAs significantly modify the pharmacokinetics and biodistribution of the ^89^Zr-mAb. Furthermore, we established that the biodistribution of HANAs can be conveniently modulated by introducing PEG polymers on the surface, facilitating customization for cancer applications. This versatile radiolabeling strategy provides a facile approach for the *in vivo* evaluation of complex nanoformulations loaded with mAbs, in a quantitative manner with high sensitivity.

## Introduction

Personalized medicine involves tailoring treatment and diagnosis to improve the therapeutic outcome of individual patients. In this context, monoclonal antibodies (mAbs) stand out in our current theranostic arsenal due to their high specificity and selectivity, enabling complex interactions with accessible targets.^1,2^ This characteristics make mAbs ideal candidates for targeting mutated oncoproteins, present in approximately 30% of human cancers.^3^ However, due to their inability to cross cell membranes and consequently target the vast array of intracellular components, their full potential remains unexplored. Nanotechnology has been proposed as an promising technology to aid mAbs overcome this biological barrier and facilitate their access to intracellular targets.^4,5^ For this purpose, certain design parameters have been stablished. For instance, particle sizes near or below 100 nm, and neutral and hydrophilic coating surfaces have been described for evading recognition by the reticulo-endothelial system.^6−8^ Specifically, PEGylation is the leading strategy as demonstrated by the increasing number of PEGylated nanoparticles (NPs) available on the market.^9,10^

The concept of intracellular mAb delivery via rationally designed nanomedicines was first explored through the development of hyaluronic acid (HA)-based nanocapsules for delivering antigasdermin B mAb.^11^ Since then, few but successful alternative mAb-delivery systems, including liposomes,^12^ micelles,^13,14^ and poly(lactic-glycolic)-based NPs^15^ have been developed. In a further effort, our group recently reported the design and characterization of HA-based nanoassemblies (HANAs) for the intracellular delivery of mAbs.^16,17^ HANAs benefit from the simplicity and safety of their composition and formulation method and remarkable mAb loading capacity.^16^ This technology was later proposed as an efficient therapy for KRAS mutant tumors, as the intracellular delivery of an antiKRAS mAb led to a reduction in tumor growth in a pancreatic cancer model.^17^

In general, the limited information on the whole-body biodistribution of these kind of nanoassemblies for either intra- or extracellular targets hinders their development and transition toward clinical products, which highlight the need to integrate efficient and simple methods to study their *in vivo* pharmacokinetics and biodistribution.^5,18^ To date, these methods have relied on fluorescence imaging that suffers from limited tissue penetration and quantification properties.^12,14,19^ Positron emission tomography (PET) is a noninvasive nuclear imaging technique that provides quantitative information with high sensitivity and outstanding tissue penetration for both animal and human studies. Among the available positron emitters, the radionuclide zirconium-89 (^89^Zr) has been widely used in clinical trials for imaging mAbs due to its convenient half-life (78.4 h) that aligns with the biological circulation of most mAbs.^20^ In this study, we report a ^89^Zr radiolabeling strategy for both PEGylated and non-PEGylated mAb-loaded HANAs. *In vivo* pharmacokinetics and biodistribution PET/CT studies in healthy animal sand *ex vivo* biodistribution were undertaken to assess the fate of the HANAs in a quantitative manner.

## Materials and Methods

### Materials

The humanized monoclonal antibody bevacizumab (BVZ) was kindly donated by mAbxience (Spain). Sodium palmitoyl hyaluronate (HAC16 30–70 kDa, degree of substitution, DS, 1–10%) was purchased from Contipro a.s. (Czech Republic). Phosphatidylcholine from soybean (Lipoid S100) and N-(carbonyl-methoxypolyethyleneglycol-_2000_)-1,2-distearoyl-*sn*-glycero-3-phosphoethanolamine (DSPE.PEG_2K_) were acquired from Lipoid GmbH (Germany). GMP grade zirconium-89 as [^89^Zr]Zr-oxalate in 1 M oxalic acid was purchased from PerkinElmer (BV Cyclotron, VU Amsterdam, NL). N1-hydroxy-N1-(5-(4-(hydroxy(5-(3-(4-isothiocyanatophenyl)thioureido)pentyl)amino)-4-oxobutanamido)pentyl)-N4-(5-(*N*-hydroxyacetamido)pentyl)succinimide (*p*-NCs-Bz-deferoxamine (DFO)) was obtained from Chematech (Dijon, France). Human serum was acquired from Sigma-Aldrich (Darmstadt, Germany). Amicon centrifuge filters (30 and 100 kDa MWCO) were provided by Merck (Darmstadt, Germany). PD-10 column filled with Sephadex G25 gel and the Superose 6 10/300 GL column were purchased from Cytiva (Marlborough, MA). Radioactivity was measured with a CRC-25R dose calibrator (Capintec) or a 1282 CompuGamma γ-counter (LKB Wallac, Finland). The 96 multiwells plate and the recombinant human VEGF_165_ were provided by Thermo Fisher Scientific. Resazurin sodium salt and Triton X-100 were provided by Merck (Darmstadt, Germany). Goat antihuman IgG HRP conjugated was purchased from Jackson Immuno Research Laboratories, Inc. 2,2′-azino-di(3-ethylbenzthiazoline sulfonic acid) (ABTS) solution was obtained from Roche (Switzerland).

### Synthesis of the Deferoxamine (DFO)-BVZ Conjugate

BVZ (5 mg) in 1 mL of PBS was mixed with 110 μL of 0.1 M Na_2_CO_3_ to achieve a solution with a pH range between 8.9 and 9.1 and thoroughly mixed by vortex. To this solution were added 20 μL of 5 mM DFO dissolved in DMSO in steps of 5 μL homogenized by vortex at room temperature (RT). The mixture was incubated at 37 °C for 30 min, and the final product was purified by centrifugal filters 30 kDa MWCO (13,000 rpm for 3 min, at least 3 cycles) and washed 3 times after resuspension to its original volume with ultrapure water following the above centrifugation settings. Finally, the purified BVZ-DFO conjugate was made up to 1 mL with ultrapure water. The success of the conjugation was evaluated by assessing ^89^Zr radiolabeling yield vs unmodified BVZ (vide infra).^21^

### Preparation of BVZ-Loaded HANAs

HANAs were prepared following a self-assembling method previously reported by our group and adapted accordingly.^16,17^ To entrap BVZ-DFO in the HANAs, the following stock solutions were prepared: HAC16 was dissolved in a 6:4 (v/v) ultrapure water: ethanol mixture, Lipoid S100, and DSPE.PEG_2K_ were dissolved in ethanol.

Briefly, non-PEGylated HANAs were prepared by adding in a one-step addition 125 μL of an aqueous solution of BVZ (final concentration of 2 mg/mL) to 500 μL of an HAC16 solution (final concentration of 2 mg/mL) under magnetic stirring at 1100 rpm and RT. Then, 50 μL of Lipoid S100 solution (final concentration of 1 mg/mL) were added drop-by-drop over the BVZ-DFO/HAC16 mixture. Thereafter, the volume was made up to 1 mL with PBS. The resulting BVZ-DFO encapsulated nanoassemblies will be named HANAs henceforth.

For PEGylated HANAs, a similar formulation method was followed. In brief, 125 μL of an aqueous solution of BVZ (final concentration of 0.5 mg/mL) was added over 500 μL of an HAC16 solution (final concentration of 0.25 mg/mL) under magnetic stirring at 1100 rpm and RT. Then, 50 μL of Lipoid S100:DSPE.PEG_2K_ solution (final concentration of 0.5 and 0.25 mg/mL, respectively) were added drop-by-drop over the BVZ-DFO/HAC16 mixture, and so-called PEG-HANAs. Finally, both types, HANAs and PEG-HANAs were concentrated via water evaporation under a N_2_ stream.

### Radiolabeling of BVZ-Loaded HANAs with ^89^Zr and Radiochemical Characterization

**Scheme 1:**
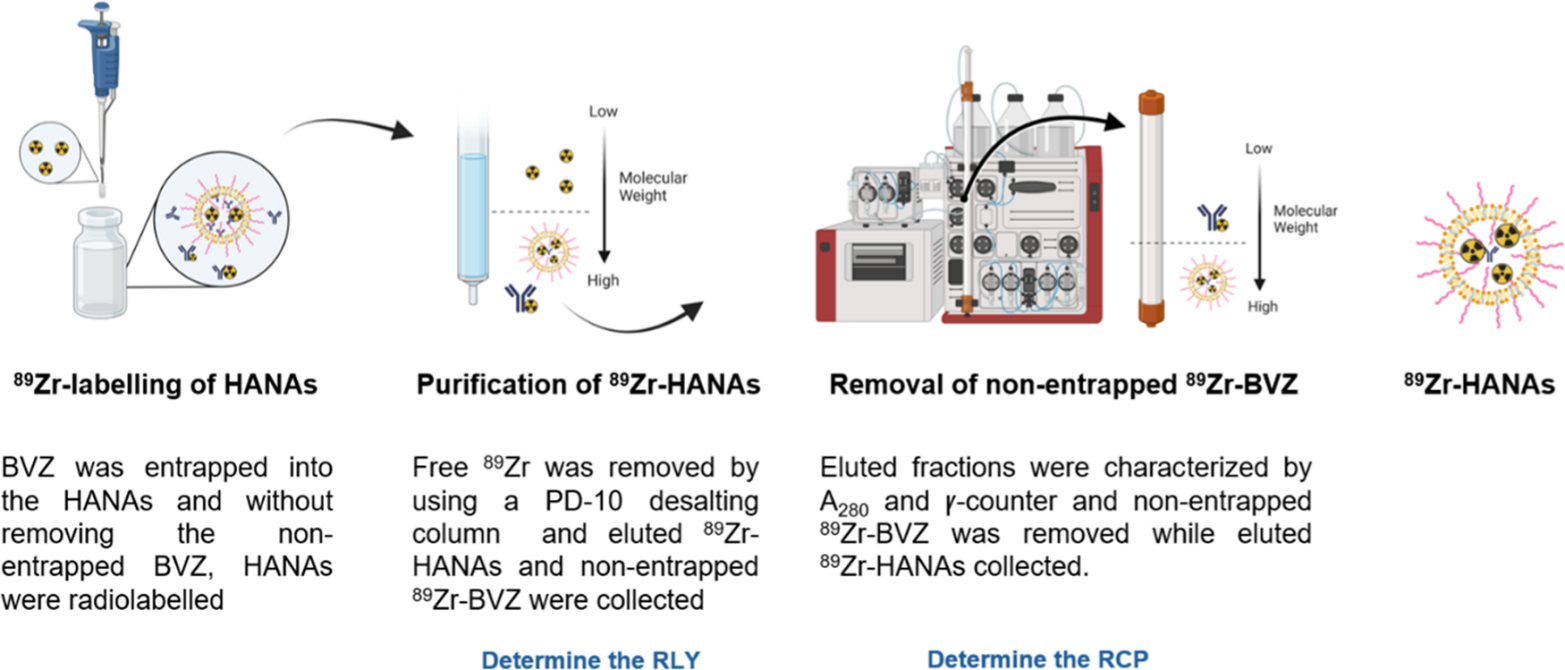
Representation of the Protocol Followed for the Purification and Radiochemical Characterization of the ^89^Zr-HANAs In brief, a mixture of ^89^Zr-HANAs, ^89^Zr-BVZ, and ^89^Zr are loaded into a PD-10 desalting column to remove free ^89^Zr. By comparison of the total activity loaded with the amount of free ^89^Zr entrapped into the column, the RLY is determined. Afterward, eluted ^89^Zr-HANAs and ^89^Zr-BVZ are loaded into a fast protein liquid chromatography system to obtain the UV/Vis profile and determine the RCP after γ-counting. RCP was determined by comparison the total activity eluted with the fractions corresponding to the ^89^Zr-HANAs. Finally, the fractions that corresponds to the pure ^89^Zr-HANAs are collected and concentrated.

^89^Zr radiolabeling of BVZ-loaded HANAs was performed as follows (Scheme 1): 200 μL of 1 M oxalic acid containing ^89^Zr were neutralized with 90 μL of 2 M sodium carbonate pH 7–8 and incubated for 3 min at RT. Then, 300 μL of 0.5 M HEPES buffer pH 7.2, 700 μL of BVZ-DFO-HANAs at 4 or 16 mg/mL, and 710 μL of 0.5 M HEPES buffer pH 7.2 were consecutively added over the previous solution and mixed at RT under horizontal agitation. After 1 h of incubation, the resulting mixture was loaded onto a PD-10 desalting column and prewashed with 20 mL of PBS, and the eluate was discarded. The ^89^Zr-labeled BVZ-loaded HANAs (^89^Zr-HANAs) and ^89^Zr-BVZ were collected from the column by the addition of 2 mL of PBS. The amount of radioactivity in the ^89^Zr-HANAs were measured in an ionization chamber and the radiolabeling yield (RLY) was calculated as
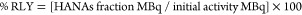
where MBq = megabecquerel.

The %RLY for free ^89^Zr-BVZ, used as a control, was determined following the same procedure. Once free ^89^Zr was removed, the eluted mixture of ^89^Zr-BVZ and ^89^Zr-HANAs were concentrated at least 4 times by using centrifugal filters of either 30 kDa or 100 kDa at 13,000 rpm for 3 min. To ensure removal of free ^89^Zr-BVZ from the ^89^Zr-HANAs, the previous concentrated mixture of ^89^Zr-BVZ and ^89^Zr-HANAs were added into a high-resolution size-exclusion chromatography column (Superose 6 10/300 GL column) in an ÄKTA size-exclusion fast protein liquid chromatography (GE Healthcare, Amersham, UK and Unicorn software) using PBS as eluent at 0.5 mL/min. Eluted fractions containing the pure ^89^Zr-HANAs, which are fractions between 7–12 mL, were collected and concentrated by centrifugal filters to achieve the desired concentration of ^89^Zr-HANAs. The eluted fractions were γ-counted and the ^89^Zr-HANAs radiochemical purity (RCP) calculated as
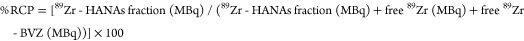
where MBq = megabecquerel.

As a control, ^89^Zr-BVZ was submitted to the same method but ÄKTA purification. All of the γ-counted fractions were compared with the UV/vis chromatogram to confirm the fractions of interest.

To study the radiochemical stability (RCS) in human serum, ^89^Zr-HANAs were incubated in human serum at 37 °C for 24, 48, and 72 h and the retained activity in the ^89^Zr-HANAs was determined following the same procedure as in the RCP. The radioactivity was measured in the γ-counter. MBq and counts per minute were correlated with an ^89^Zr calibration curve (Figure S1).

All of the radiolabeling processes and characterization described for ^89^Zr-HANAs were applied to ^89^Zr-PEG-HANAs.

### Physicochemical Characterization

Particle size, polydispersity index (PDI), and ζ-potential were measured by photon correlation spectroscopy using a Malvern Zeta-Sizer (NanoZS, ZEN 3600, Malvern Instruments, U.K.). Prior to the measurements, the samples were diluted in PBS. A cumulant analysis was used to obtain the hydrodynamic diameter calculated as the Z-average value based on the intensity distribution.

Radiochemical parameters were determined by an γ-counter or ionization chamber. MBq and counts per minute were correlated with an ^89^Zr calibration curve (Figure S1).

### Association Efficiency (AE) and Loading Capacity (LC) of the HANAs

BVZ AE % of the nonradiolabeled HANAs was quantified using an enzyme-linked immunosorbent assay (ELISA) following a previously described method.^16^ A Beckman Coulter (optime L90K) ultracentrifuge equipped with a Beckman type 70.1 Ti rotor was used to isolate the HANAs (35,000 rpm, 1.5 h, 15 °C). The amount of free BVZ in the supernatant was recovered and quantified. A control solution of BVZ at 3.2 mg/mL was treated under the same conditions and subsequently quantified.

A 96-multiwell plate was coated with 0.005 μg of antigen/well (i.e., recombinant human VEGF_165_) at a concentration of 0.05 μg/mL (100 μL/well). After incubation overnight at 4 °C, the plate was washed with Tween 20 at 0.05% (v/v) in PBS, pH 7.4. Then, a blocking step was performed to avoid unspecific bindings with 300 μL/well of blocking buffer (2% v/w of dry milk powder prepared in washing buffer) for 2 h at 37 °C under orbital shaking at 300 rpm, followed by another washing step. A BVZ calibration curve (from 1000 ng/mL to 1.50 ng/mL) and the BVZ retrieved from the supernatant were diluted in PBS accordingly and loaded onto the described antigen-coated plate. After incubation at 37 °C for 1 h, the plate was subjected to a washing step. Afterward the secondary goat antihuman HRP antibody was added at a concentration of 0.08 μg/mL and incubated for 1 h at 37 °C. After washing, 50 μL/well of ABTS (detection substrate) was added. The plate was incubated at RT for 25 min and samples absorbances were measured at 405 nm using the Biotek Synergy H1 microplate reader (Gen5 software). The AE and LC were calculated as follows:
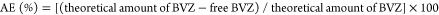

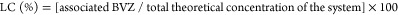
where in the total theoretical concentration includes the amount of HAC16, Lipoid S10 and/or DSPE.PEG_2K_, and BVZ used in the HANAs formation.

### Colloidal Stability upon Storage of ^89^Zr-PEG-HANAs

The colloidal stability of the ^89^Zr-PEG-HANAs was evaluated under storage conditions at 4 °C at selected time points (0, 2, and 9 days). Their colloidal properties were studied based on the physicochemical characteristics (particle size and PDI) and the amount of radioactivity in the particles, the released mAb and the detached free ^89^Zr (i.e. ^89^Zr-PEG-HANAs, ^89^Zr-BVZ or free ^89^Zr). At each time point, 0.5 mg/mL of ^89^Zr-PEG-HANAs were injected into the ÄKTA equipment using the conditions described above. The eluted fractions were collected, and the radioactivity was measured in a γ-counter. The radioactivity in the ^89^Zr-PEG-HANAs, ^89^Zr-BVZ, and free ^89^Zr was correlated with the total activity and expressed as percentage of ^89^Zr in each fraction. These studies were not conducted in serum as free ^89^Zr coelutes with serum proteins showing similar retention time than ^89^Zr-BVZ, which makes the peaks corresponding to ^89^Zr-BVZ and free ^89^Zr undistinguishable.

### Cell Viability Assays

To evaluate the cytotoxicity profile of the ^89^Zr-PEG-HANAs, an Alamar Blue assay was performed on the A549 human lung cancer cell line. For this purpose, 7500 cells/well were seeded in a 96-well plate and incubated overnight at 37 °C with 5% CO_2_. Dulbecco’s Modified Eagle Medium (DMEM) (high glucose) containing 10% fetal bovine serum, penicillin, and streptomycin was used as cell culture media. When the confluence was 70–80%, the cell culture media was discarded and replaced with 100 μL/well of increasing concentrations of blank and PEG-HANAs. After 24 h of exposure, the media was removed and 100 μL/well of the 1X resazurin reagent diluted in cell culture media was added and incubated for 40 min. Fluorescence intensity as an indicator of the number of live cells is proportional to the reduction of resazurin to resorufin. Fluorescence values were recorded in a microplate reader (Promega, Madison, WI), with the excitation/emission intensity at 525/580–640 nm. Cells treated with cell culture media were used as positive controls. Triton X-100 0.5% (v/v) in cell culture media was added to cells and used as the negative control. The percentage of cell viability was calculated, after subtracting the values from the negative control, as the fluorescence of the samples divided by the positive control.

The cytotoxicity profile of ^89^Zr-PEG-HANAs was determined following the above-described method. In this case, cells were exposed to increasing concentrations of the ^89^Zr-PEG-HANAs (*i.e*., 0.005, 0.05, and 0.5 mg/mL) for 4 h. This time was selected as it has been demonstrated to be sufficient for uptake evaluation.^16^ To discard cytotoxicity associated to the mAb,^22,23^^89^Zr-BVZ was used as control. The percentage of cell viability was calculated as described above. The radiolabeling process of the HANAs requires several concentration and purification procedures, in order to calculate the mass of the formulations be used in the study, the synthesis yield of the HANAs was determined gravimetrically. 40 μL of purified ^89^Zr-PEG-HANAs and ^89^Zr-BVZ were added to a glass vial and dried by evaporation at 100 °C. After that, the vial was weighted, and the final concentration was calculated after subtracting the weight of the empty vial.

cell viability (%) = sample fluorescence – negative control fluorescence/positive control fluorescence.

### *In Vivo* PET/CT Imaging of ^89^Zr-HANAs in Healthy Mice

Animal imaging studies were ethically reviewed and carried out in accordance with the Animals (Scientific Procedures) Act 1986 (ASPA) UK Home Office regulations governing animal experimentation. *In vivo* imaging was conducted in healthy 8 week old C57BL/6 mice. Blood samples were withdrawn at 25 min and 1, 2, 5.5, 20, 25, and 72 h. PET/CT images were recorded at 90 min, 24 and 72 h after intravenous injection of the NP. Animals were anesthetized with isoflurane (2–3% in oxygen) and the radiolabeled NPs (1.5–3 MBq) intravenously administrated 1 h before the PET acquisition (1:5 coincidence mode; 5 ns coincidence time window). PET was recorded for 30 min and then a semicircular CT scan was performed. Animal body temperature was maintained at 37 °C, and the respiratory rate was monitored during the imaging protocol. PET/CT images were reconstructed using Tera-Tomo 3D reconstruction (400–600 keV energy window, 1:3 coincidence mode, 4 iterations, and 6 subsets) at a voxel size of (0.4 × 0.4 × 0.4) mm^3^ and corrected for attenuation, scatter, and decay. Blood half-life was calculated as follows:
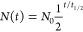
where *N*(*t*) = remaining quantity after time, *t*; *N*_0_ = initial quantity; *t*_1/2_ = half-life.

### Statistical Analysis

Statistical analyses were calculated using GraphPad Prism software. Data were analyzed by unpaired *t*-test and 2-way ANOVA followed by Fisher’s LSD test. *p ≤ 0.05, **p ≤ 0.01, ***p ≤ 0.001, and ****p ≤ 0.0001 were considered statistically significant. The statistical analysis details are provided in the corresponding figure legends.

## Results and Discussion

### Composition and Properties of BVZ-Loaded HANAs

We engineered a singular delivery system, the HANAs technology, which combines two key components, HAC16 and phosphatidylcholine (Lipoid S100), with a high capacity of interaction with the mAb cargo. While the mAb interacts with the HAC16 by hydrophobic and anionic interactions, Lipoid S100 plays a key role in the induction of assembling the three components. This selection led to the entrapment of high amounts of mAb through a one-step assembling process, leading to a nanoassembly of tunable physicochemical properties.^16,17^ With this technology, we have demonstrated the capacity of the HANAs technology for the intracellular delivery of mAbs.^16,17^ Indeed, this technology have been proposed as an efficient therapy for KRAS mutant tumors, as the intracellular delivery of an antiKRAS mAb led to a reduction in tumor growth in a pancreatic cancer model.^17^

In this work, we have selected two types of HANAs loaded with the model mAb, BVZ, with different surface compositions and particle sizes in order to evaluate their *in vivo* performance. Our previous data showed that the physicochemical and chemical properties of HANAs could be tunned based on the proportion of the components of HANAs, as evidenced by computational modeling.^16^ Specifically, we have chosen BVZ-loaded non-PEGylated HANAs (HANAs) and PEGylated HANAs (PEG-HANAs), with particle sizes of 160 and 80 nm, respectively and PDI < 0.3 (Figure 2A). AE and LC were superior to 67 and 22%, w/w, respectively (Figure 2A). These are outstanding results compared to other mAb-loaded nanocarriers.^22−24^ The tunable particle size and high AE have been attributed to the formulation method, the low concentrations of starting materials for PEG-HANAs, and the high affinity of mAb with amphiphilic HA and Lipoid S100, among others. This hypothesis was demonstrated by computational modeling and further supported by the analysis of the structural configuration by the orthogonal sizing techniques cryogenic transmission electron microscopy, asymmetrical flow field-flow fractionation, and small-angle X-ray scattering.^16^

### Radiolabeling of BVZ-Loaded HANAs with ^89^Zr

**Figure 1:**
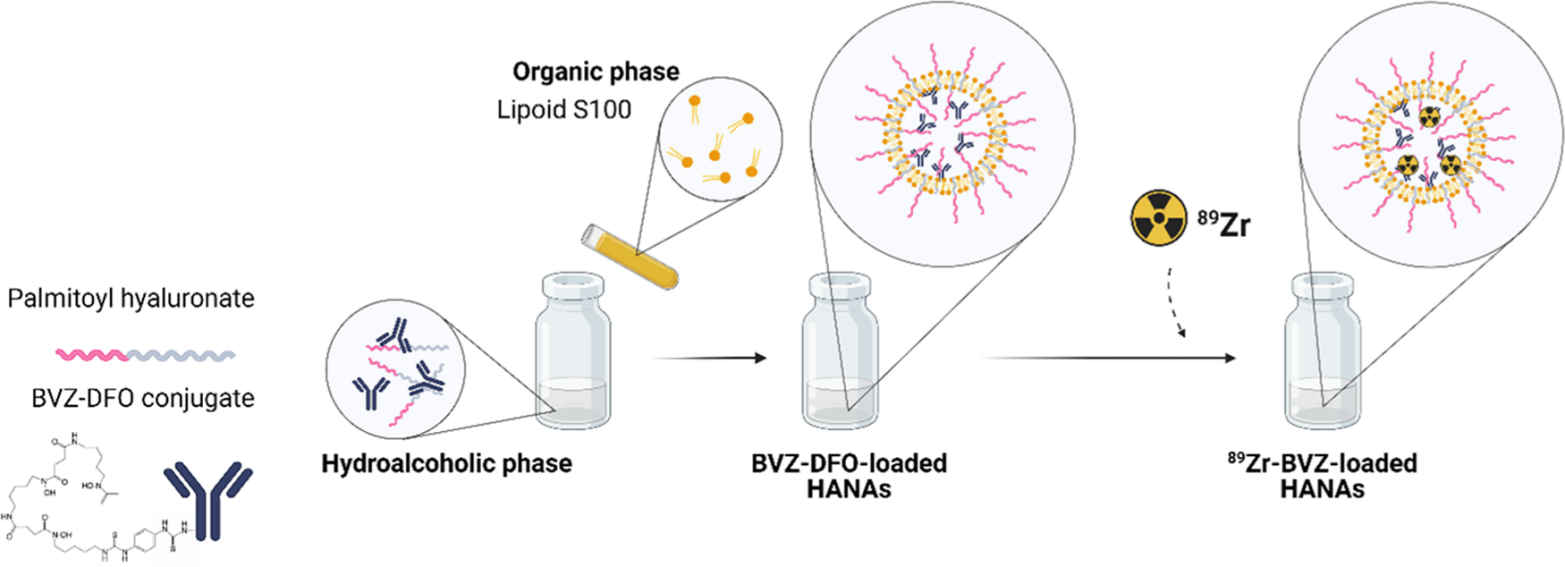
Schematic representation of the formulation and radiolabeling method used for the ^89^Zr-labeling of the HANAs. As indicated, the BVZ-DFO conjugate is entrapped into the NPs and, afterward, the entrapped mAb is radiolabeled with ^89^Zr.

The radiolabeling reaction was performed by first conjugating the DFO chelator to the mAb. BVZ was selected as a model mAb, anticipating the implementation of a similar approach for other mAbs. This was followed by a reaction with [^89^Zr]Zr-oxalate after the entrapment of the BVZ-DFO within the NPs (Figure 1). It is important to consider that the suitability of the radiolabeling method for *in vivo* studies will be determined by the capacity to radiolabel the HANAs while preserving the physicochemical properties of the particles and achieving a high RCP and stability (*vide infra*).

**Figure 2:**
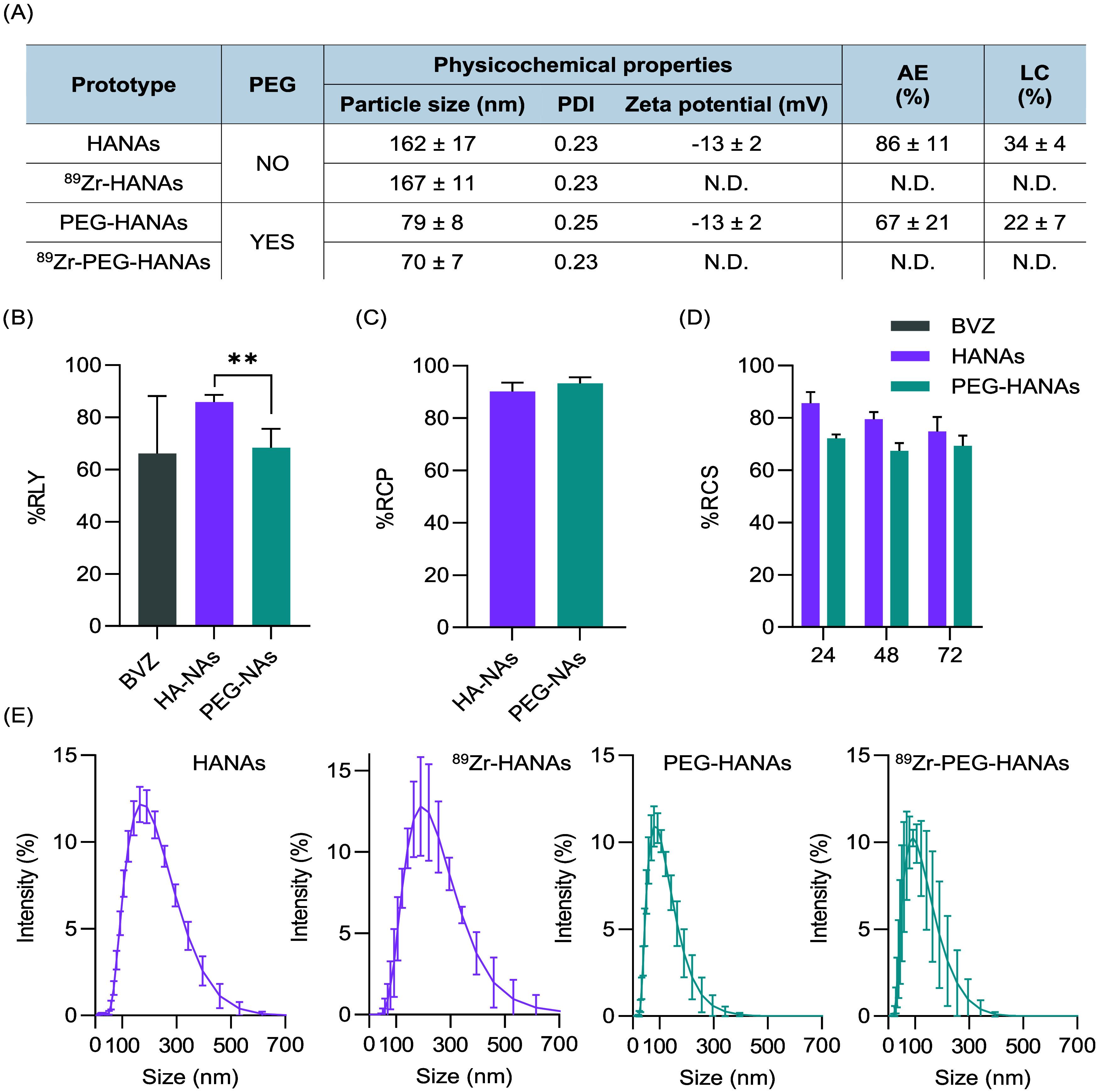
Radiochemical characterization of ^89^Zr-BVZ-loaded HANAs and ^89^Zr-BVZ-loaded PEG-HANAs. (A) Table with the physicochemical properties, association efficiency (AE, %) and loading capacity (LC, %) of the nonradiolabeled and radiolabeled HANAs (ND = not determined). The system with a PEG component was noted. Data are expressed as mean ± SD, *n* ≥ 3. (B) Radiolabeling yield (RLY) of ^89^Zr-BVZ, ^89^Zr-HANAs, and ^89^Zr-PEG-HANAs (*n* ≥ 3). (C) Radiochemical purity (RCP) of 89Zr-HANAs and 89Zr-PEG-HANAs (*n* = 3), and (D) radiochemical stability (RCS) in human plasma at 37 °C for 24, 48, and 72 h (*n* = 2). (E) Hydrodynamic size of the HANAs measured by DLS before and after the ^89^Zr radiolabeling process (*n* ≥ 3). Data are expressed as mean ± SD. Statistical analysis in RLY and RCP was done using unpaired *t*-test, **p < 0.01.

Briefly, BVZ was conjugated with DFO via reaction with an amine-reactive DFO-thioisocyanate (DFO-NCS) to form a stable thiourea bond. This is a well-stablished strategy for the radiolabeling of mAb with ^89^Zr.^21^ In order to radiolabel the BVZ-loaded HANAs, DFO-BVZ was entrapped in the HANAs. Afterward, the DFO-BVZ-HANAs were incubated with neutralized [^89^Zr]Zr-oxalate at RT for 1 h and purified by size-exclusion chromatography to provide the ^89^Zr-labeled BVZ-HANAs (^89^Zr-HANAs and ^89^Zr-PEG-HANAs). As control of the radiolabeling reaction, nonencapsulated DFO-BVZ was submitted to the same radiolabeling process (^89^Zr-BVZ). Moderate-to-high radiolabeling yields (RLYs) of 66 ± 22, 86 ± 3, and 68 ± 11% were obtained for ^89^Zr-BVZ, ^89^Zr-HANAs, and ^89^Zr-PEG-HANAs, respectively (Figure 2B). The superior RLY for ^89^Zr-HANAs over ^89^Zr-PEG-HANAs suggest that the structural organization and composition of the PEG-HANAs (i.e., PEG outer shell) may limit the diffusion of the ^89^Zr toward the entrapped DFO-BVZ at RT. The incorporation of radiometals across a lipid bilayer without the need of a transport chelator (i.e., an ionophore) have been previously described for PEGylated liposomes, showing that the optimal radiometal loading rates were achieved at 55 °C.^25,26^ In our case, reactions were conducted at RT which may lead to decreased RLYs, but preserving the biological activity of the mAb.^27^

The RCP was determined for both prototypes (Figure 2C). The isolation of mAb-loaded NPs from nonentrapped mAbs represents a significant challenge, primarily due to the absence of methods enabling simultaneous separation and quantification. While an array of methods are available for mAb isolation and purification, challenges arise when implementing them for the isolation and quantification of mAb-loaded NPs. These challenges include difficulties isolating the NPs from the mAb efficiently, quantifying low mAb concentrations, the matrix complexity, and the difficulty in preserving the physicochemical properties of the NPs during separation. In this study, a size-exclusion fast protein liquid chromatography system was employed to isolate the nonentrapped mAb from the mAb-loaded NPs. RCP values >90% were found, which demonstrate the successful removal of unlabeled ^89^Zr and nonentrapped ^89^Zr-BVZ from the mAb-loaded NPs. In parallel, the physicochemical properties of both ^89^Zr-HANAs and ^89^Zr-PEG-HANAs were evaluated revealing nonsignificant changes (Figure 2A). Additionally, the RCS of ^89^Zr-HANAs in human serum was evaluated (Figure 2D). The RCS determines whether the radionuclide is detached from the particle under physiological conditions. A high RCS is essential to avoid the misinterpretation of PET imaging, where the signal of the detached radionuclide could be interpreted as the biodistribution of the NPs. In our case, the RCS varied from 86 ± 4 and 72 ± 2% at 24 h to 75 ± 6 and 69 ± 4% for ^89^Zr-HANAs and ^89^Zr-PEG-HANAs after 72 h without significant differences between prototypes. Although these values are lower than other ^89^Zr-mAbs and derivatives,^28,29^ they were considered adequate for further PET imaging interpretation, based on the significantly different biodistribution of the various individual components. To the best of our knowledge, this is the first reported radiolabeling strategy for tracking polymeric NPs through encapsulated ^89^Zr-mAb that provides suitable radiochemical properties and reproducible procedures, without altering the physicochemical properties of the NPs. Two previous reports have described a similar radiolabeling strategy for BVZ-loaded albumin-based NPs and antiCTLA-4 mAb-loaded PEGylated liposomes with ^99m^Tc.^30,31^ However, the radiolabeling procedure altered the physicochemical properties of the particles in one case and was not reported it in the other.

### Colloidal Stability upon the Storage of ^89^Zr-HANAs

**Figure 3:**
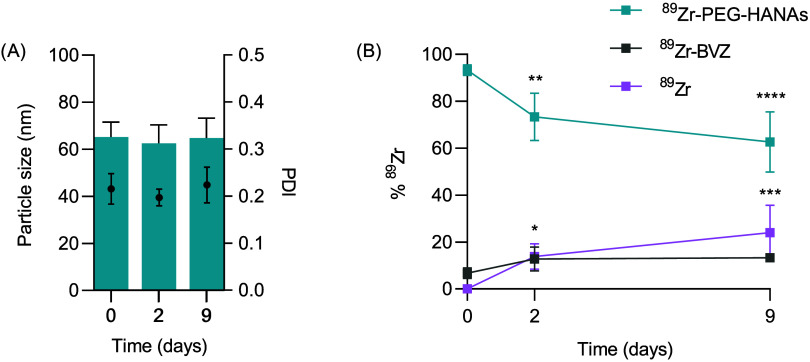
Stability in suspension of 89Zr-PEG-HANAs at 4 °C for 9 days. (A) Evolution of the particle size (bars) and PDI (dots) of the prototypes during storage for up to 9 days. Statistical analysis of the particle size was done using unpaired *t*-test. (B) Cumulative percentage of 89Zr present in each fraction, i.e., PEG-HANAs, BVZ, and free overtime at the indicated time point for 89Zr-PEG-HANAs. Statistical analysis between day 0 and the following days for each fraction was done using 2-way ANOVA followed by Fisher’s LSD test, *p < 0.05, **p < 0.01, ***p < 0.001, and ****p < 0.0001. Data are expressed as mean ± SD, *n* = 3.

The particle size, polydispersity index (PDI), and the amount of released ^89^Zr and ^89^Zr-BVZ were studied to evaluate the colloidal stability at 4 °C for 9 days in PBS. We selected PEG-HANAs for this study as the most promising candidate given their optimal particle size for tumor accumulation. The data shown in Figure 3A revealed minor variations in terms of particle size and PDI for at least 9 days. Regarding ^89^Zr release, a significant decrease of ^89^Zr complexed to the HANAs was observed over time (when compared to HANAS prior storage, Figure 3B). In turn, a significant increase in the released of free ^89^Zr was observed, while the amount of ^89^Zr-BVZ remained stable from day 2 to 9. This behavior is comparable to the RCS observed in human plasma, suggesting that the formation of a protein corona did not alter the ^89^Zr release kinetics.^32^ Our findings are in line with other reported storage stabilities in terms of ^89^Zr leakage for other mAbs.^33^

### Cell Viability

**Figure 4:**
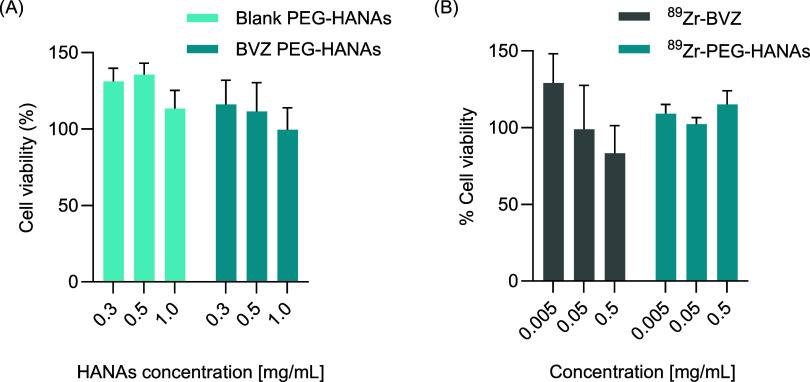
*In vitro* metabolic activity of PEG-HANAs on the A549 cell line. Cell viability (%) of (A) nonencapsulated blank and PEG-HANAs after 24 h of incubation at 0.3, 0.5, and 1 mg/mL and (B) of ^89^Zr-BVZ and ^89^Zr-PEG-HANAs after 4 h of incubation at 0.005, 0.05, and 0.5 mg/mL. ^89^Zr activities (Bq/well) of 2.2 × 10^5^ (^89^Zr-BVZ) and 1.0 × 10^5^ (^89^Zr-PEG-HANAs) were tested at the highest total concentration (i.e., 0.5 mg/mL). Total concentration (mg/mL) was calculated considering the dry mass of the final product after the radiolabeling. Data are expressed as mean ± SEM, *n* ≥ 3.

The toxicity of HANAs could be affected after the radiolabeling due to the presence of ionizing radiation. In fact, radiotoxicities caused by ionizing radiation can be directly caused by radiochemical impurities or side products, or indirectly due to water radiolysis.^34^ Aiming to evaluate the potential radiotoxicity of the leading candidate (^89^Zr-PEG-HANAs), a metabolic activity assay was carried out in the nonsmall cell lung cancer A549 line. We chose nonsmall cell lung cancer as it has been linked to mutated intracellular oncoproteins,^35,36^ suggesting it would benefit from an improved intracellular delivery of mAbs. Indeed, in a previous report, we have demonstrated the capacity of the HANAs to aid the intracellular delivery of a relevant antiKRAS mAb and further engagement of the target KRAS, one of the most prevalent mutations in cancer.^17^ We first evaluated the cell viability at different concentrations of nonencapsulated PEG-HANAs (blank) and the BVZ encapsulated PEG-HANAs without radioactivity to study the toxicity induced by the components of the HANAs (Figure 4A). A low-toxicity profile for up to 1 mg/mL of the nonradiolabeled HANAs was observed, similar to the results previously obtained after incubation of HANAs in the CMT167 lung cancer cell line, confirming the biocompatible profile of the NPs.^16^ Then, the radioactive counterpart ^89^Zr-PEG-HANAs was evaluated using ^89^Zr-BVZ as a control. After 4 h of treatment, increasing concentrations of ^89^Zr-PEG-HANAs or ^89^Zr-BVZ did not modify the cell viability within the range of tested activity per cell (Figure 4B). Hence, the incorporation of ^89^Zr into HANAs did not affect their cytotoxicity profile. Cell viability greater than 100% after incubation with the particles can be attributed to experimental variability, as values are calculated relative to the negative control. Additionally, since cell viability was assessed using a metabolic assay, higher values could indicate increased metabolic activity triggered by PEG-HANAs uptake.

### The Biodistribution of the mAb is Modified through Its Entrapment into the HANAs

Aiming to evaluate the *in vivo* performance of the HANAs, PET/CT imaging and biodistribution studies were conducted in healthy C57BL/6 mice. To compare the capacity of the NPs to modify the biodistribution profile of the mAb, nonentrapped radiolabeled mAb (^89^Zr-BVZ) and the BVZ-loaded HANAs, ^89^Zr-HANAs and ^89^Zr-PEG-HANAs, were evaluated up to 72 h.

**Figure 5:**
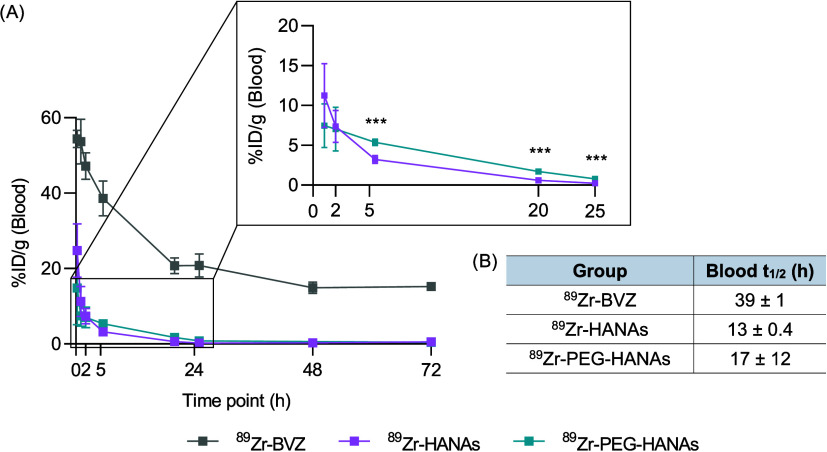
Percentage of the injected dose per gram (%ID/g) of blood in healthy mice for 72 h. (A) Inset of the first 25 h comparing the circulation time in blood of ^89^Zr-HANAs and ^89^Zr-PEG-HANAs. (B) Table indicates the blood half-life (*t*_1/2_) after 72 h of circulation. Data are expressed as mean ± SD, *n* = 4. Statistical analysis was done using unpaired *t*-test, ***p < 0.001.

The pharmacokinetics demonstrated a rapid clearance of ^89^Zr-HANAs and ^89^Zr-PEG-HANAs from the bloodstream (Figure 5). After 2 h in circulation, the % ID/g declined by 70% for ^89^Zr-HANAs and 53% for ^89^Zr-PEG-HANAs, indicating an extended circulation due to the presence of PEG. Additionally, the mAb demonstrated prolonged circulation, with only a marginal decrease of 13% after 2 h, corresponding to a half-life of 39 ± 1 h, consistent with previously reported circulation times with the same BVZ.^37^^89^Zr-PEG-HANAs demonstrated a significantly higher circulation time from 5 to 25 h than ^89^Zr-HANAs, attributed to the stealth effect of the PEG shell. Although statistically significant, the half-life of ^89^Zr-PEG-HANAs (17 ± 12 h) was superior to ^89^Zr-HANAs (13 ± 0.4 h), further supporting the stealth hypothesis. Indeed, the PEG effect was observed at a theoretical molar density of 12 lipid mol %, which is higher than the PEG densities (0.5–5%) that have demonstrated efficient passive accumulation.^31,38^ The fact that high PEG densities did not led to a superior circulation time could be explained by the PEG desorption kinetics,^39^ which determines the nature of the protein corona adsorbed and therefore, the tissue distribution. This effect needs to be studied in detail, taking into account the unique characteristics of each formulation and the influence of the mAb presence within the NP.

**Figure 6:**
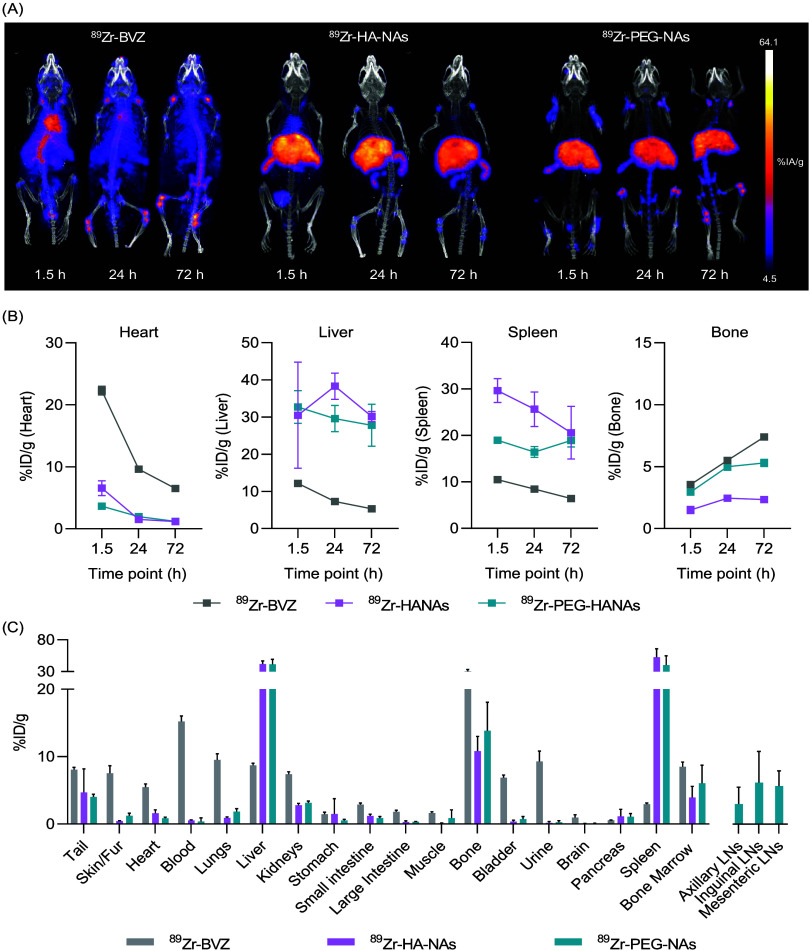
PET/CT imaging distribution of ^89^Zr-BVZ, ^89^Zr-HANAs, and ^89^Zr-PEG-HANAs in healthy C57BL/6J mice. (A) PET/CT maximum intensity projections (MIP) after iv administration of ^89^Zr-BVZ, 89Zr-HANAs, and ^89^Zr-PEG-HANAs (C carotids; H heart; A aorta; B bone; L liver; S spleen; Bl bladder). (B) PET quantification expressed as percentage of the injected dose per gram (%ID/g) in heart, liver, spleen, and bone at 1.5, 24, and 72 h after i.v. administration of ^89^Zr-BVZ, ^89^Zr-HANAs, and ^89^Zr-PEG-HANAs. Data are expressed as mean ± SD. (C) Organ uptake by *ex vivo* γ-counting represented as %ID/g of tissue at 72 h. Statistical comparison of the organs of interest (liver and spleen) was done following an unpaired *t*-test among HANAs. LNs are lymph nodes. Error bars represent the mean ± SD, *n* = 4.

PET/CT images were consistent with the pharmacokinetics in blood, revealing a very different biodistribution profile of the HANAs compared to the mAb alone. As expected, ^89^Zr-BVZ remained in blood for long periods, while ^89^Zr-HANAs and ^89^Zr-PEG-HANAs prototypes accumulated in the liver and spleen (Figure 6A). Intravenous administration of neutralized [^89^Zr]ZrCl_4_ as secondary control showed a very distinct biodistribution compared to that of ^89^Zr-BVZ and the radiolabeled compounds confirming their high RCP and RCS (Figure S3). PET quantification in main organs at the imaging time points revealed high accumulation in liver and spleen, which is in consonance with the *in vivo* pattern of most polymer-based NPs.^40,41^ Moreover, ^89^Zr-PEG-HANAs exhibited lower liver and spleen uptake after 24 h compared to ^89^Zr-HANAs, but similar uptake after 72 h, reflecting the higher circulation of the PEGylated nanoparticles (Figure 6B). In contrast, ^89^Zr-BVZ showed a higher PET signal in the heart, related to the higher blood circulation, and significantly lower uptake in the liver and spleen (Figure 6B). *Ex vivo* γ-counter biodistribution after 72 h revealed a high uptake of ^89^Zr-PEG-HANAs in primary and secondary lymph nodes. Unfortunately, the lymph nodes of the mice injected with ^89^Zr-BVZ and ^89^Zr-HANAs were not collected. This was due to the lack of lymph node uptake observed by PET imaging. Further repeat experiments are needed to establish the lymph node uptake values in these groups. We hypothesize that the lymph node uptake with ^89^Zr-PEG-HANAs was a result of the smaller particle size. Indeed, the lymph node accumulation for nanoplatforms ranging from 10 to 100 nm have been widely described.^42,43^ Moreover, ^89^Zr-BVZ showed a higher bone uptake, presumably due to a higher presence of free ^89^Zr. Long-term detachment of ^89^Zr from radiolabeled mAbs is common when using DFO complexation.^44^ However, in the radiolabeled HANAs, this detachment is reduced because BVZ is encapsulated within the NPs, minimizing its exposure to blood proteins.

## Conclusions

Understanding the pharmacokinetics and biodistribution of delivery nanoplatforms designed to modify the biodistribution profile of naked mAbs is complicated but key for their efficient clinical translation. Here, we introduce a straightforward and efficient method for the radiolabeling of mAb-loaded HANAs with a relatively long half-life positron emitter, ^89^Zr. The radiolabeled formulations exhibited good radiochemical properties for *in vivo* applications in terms of radiochemical stabilities and purity regardless of the HANAs composition and mAb concentration, all while preserving the physicochemical properties of the NPs. Notably, no other examples have been reported for the quantitative evaluation of mAb-loaded NPs using PET/CT imaging. Understanding the factors that affect the circulation time, such as surface PEGylation of NPs, enables the customization of NPs to meet the specific oncological applications. Our radiolabeling strategy allows one to accurately track the fate of NPs loaded with mAbs and presumably, other clinically relevant mAbs. The versatility of this approach opens new avenues for the quantitative assessment of complex nanoformulations loaded with mAbs, providing a powerful tool for evaluating drug delivery systems with high sensitivity and accuracy. Future experiments could involve their evaluation in tumor-bearing animal models to further elucidate the genuine potential of this formulation in cancer nanotheranostics.

## Supporting Information Available

Calibration curve of ^89^Zr, UV/vis and radioactivity profile of HANAs, and PET/CT images of [^89^Zr]ZrCl_4_ in 9 weeks old C57BL/6J mice. (PDF)

## References

[ref1] Carter P. J.; Lazar G. A. Next Generation Antibody Drugs: Pursuit of the “High-Hanging Fruit.. Nat. Rev. Drug Discovery 2018, 17 (3), 197–223. 10.1038/nrd.2017.227. 29192287

[ref2] Slastnikova T. A.; Ulasov A. V.; Rosenkranz A. A.; Sobolev A. S. Targeted Intracellular Delivery of Antibodies: The State of the Art. Front. Pharmacol. 2018, 9, 1208 10.3389/fphar.2018.01208. 30405420 PMC6207587

[ref3] Cox A. D.; Fesik S. W.; Kimmelman A. C.; Luo J.; Der C. J. Drugging the Undruggable RAS: Mission Possible?. Nat. Rev. Drug Discovery 2014, 13, 828 10.1038/nrd4389. 25323927 PMC4355017

[ref4] Durán-Lobato M.; López-Estévez A. M.; Cordeiro A. S.; Dacoba T. G.; Crecente-Campo J.; Torres D.; Alonso M. J. Nanotechnologies for the Delivery of Biologicals: Historical Perspective and Current Landscape. Adv. Drug Delivery Rev. 2021, 176, 113899 10.1016/j.addr.2021.113899. 34314784

[ref5] López-Estévez A. M.; Lapuhs P.; Pineiro-Alonso L.; Alonso M. J. Personalized Cancer Nanomedicine: Overcoming Biological Barriers for Intracellular Delivery of Biopharmaceuticals. Adv. Mater. 2024, 36, 2309355 10.1002/adma.202309355. 38104275

[ref6] Sykes E. A.; Chen J.; Zheng G.; Chan W. C. W. Investigating the Impact of Nanoparticle Size on Active and Passive Tumor Targeting Efficiency. ACS Nano 2014, 8 (6), 5696–5706. 10.1021/nn500299p. 24821383

[ref7] Alexis F.; Pridgen E.; Molnar L. K.; Farokhzad O. C. Factors Affecting the Clearance and Biodistribution of Polymeric Nanoparticles. Mol. Pharmaceutics 2008, 5 (4), 505–515. 10.1021/mp800051m. PMC2663893 18672949

[ref8] He C.; Hu Y.; Yin L.; Tang C.; Yin C. Effects of Particle Size and Surface Charge on Cellular Uptake and Biodistribution of Polymeric Nanoparticles. Biomaterials 2010, 31 (13), 3657–3666. 10.1016/j.biomaterials.2010.01.065. 20138662

[ref9] Gao Y.; Joshi M.; Zhao Z.; Mitragotri S. PEGylated Therapeutics in the Clinic. Bioeng. Transl. Med. 2024, 9 (1), e10600 10.1002/btm2.10600. 38193121 PMC10771556

[ref10] López-Estevez A. M.; Gref R.; Alonso M. J. A Journey through the History of PEGylated Drug Delivery Nanocarriers. Drug Delivery Transl. Res. 2024, 14 (8), 2026–2031. 10.1007/s13346-024-01608-8. PMC11208220 38796665

[ref11] Molina-Crespo Á.; Cadete A.; Sarrio D.; Gámez-Chiachio M.; Martinez L.; Chao K.; Olivera A.; Gonella A.; Díaz E.; Palacios J.; Dhal P. K.; Besev M.; Rodríguez-Serrano M.; García Bermejo M. L.; Triviño J. C.; Cano A.; García-Fuentes M.; Herzberg O.; Torres D.; Alonso M. J.; Moreno-Bueno G. Intracellular Delivery of an Antibody Targeting Gasdermin-B Reduces HER2 Breast Cancer Aggressiveness. Clin. Cancer Res. 2019, 25 (15), 4846–4858. 10.1158/1078-0432.CCR-18-2381. 31064780

[ref12] Deng H.; Song K.; Zhao X.; Li Y.; Wang F.; Zhang J.; Dong A.; Qin Z. Tumor Microenvironment Activated Membrane Fusogenic Liposome with Speedy Antibody and Doxorubicin Delivery for Synergistic Treatment of Metastatic Tumors. ACS Appl. Mater. Interfaces 2017, 9 (11), 9315–9326. 10.1021/acsami.6b14683. 28244731

[ref13] Rafael D.; Montero S.; Carcavilla P.; Andrade F.; German-Cortés J.; Diaz-Riascos Z. V.; Seras-Franzoso J.; Llaguno M.; Fernández B.; Pereira A.; Duran-Lara E. F.; Schwartz S.; Abasolo I. Intracellular Delivery of Anti-Kirsten Rat Sarcoma Antibodies Mediated by Polymeric Micelles Exerts Strong In Vitro and In Vivo Anti-Tumorigenic Activity in Kirsten Rat Sarcoma-Mutated Cancers. ACS Appl. Mater. Interfaces 2023, 15 (8), 10398–10413. 10.1021/acsami.2c19897. 36795046

[ref14] Chen P.; Yang W.; Hong T.; Miyazaki T.; Dirisala A.; Kataoka K.; Cabral H. Nanocarriers Escaping from Hyperacidified Endo/Lysosomes in Cancer Cells Allow Tumor-Targeted Intracellular Delivery of Antibodies to Therapeutically Inhibit c-MYC. Biomaterials 2022, 288, 121748 10.1016/j.biomaterials.2022.121748. 36038419

[ref15] Jiang G.; Huang Z.; Yuan Y.; Tao K.; Feng W. Intracellular Delivery of Anti-BCR/ABL Antibody by PLGA Nanoparticles Suppresses the Oncogenesis of Chronic Myeloid Leukemia Cells. J. Hematol. Oncol. 2021, 14 (1), 139 10.1186/s13045-021-01150-x. 34488814 PMC8422775

[ref16] López-Estévez A. M.; Zhang Y.; Medel M.; Arriaga I.; Sanjurjo L.; Huck-Iriart C.; Abrescia N. G. A.; Vicent M. J.; Ouyang D.; Torres D.; Alonso M. J. Engineering Hyaluronic Acid-Based Nanoassemblies for Monoclonal Antibody Delivery – Design, Characterization, and Biological Insights. Nano Res. 2024, 17, 9111 10.1007/s12274-024-6826-8.

[ref17] López-Estévez A. M.; Sanjurjo L.; Turrero Á.; Arriaga I.; Abrescia N. G. A.; Poveda A.; Jiménez-Barbero J.; Vidal A.; Torres D.; Alonso M. J. Nanotechnology-Assisted Intracellular Delivery of Antibody as a Precision Therapy Approach for KRAS-Driven Tumors. J. Controlled Release 2024, 373, 277–292. 10.1016/j.jconrel.2024.07.032. 39019086

[ref18] Pellico J.; Gawne P. J.; De Rosales R. T. M. Radiolabelling of Nanomaterials for Medical Imaging and Therapy. Chem. Soc. Rev. 2021, 50 (5), 3355–3423. 10.1039/d0cs00384k. 33491714

[ref19] Chung J. E.; Tan S.; Gao S. J.; Yongvongsoontorn N.; Kim S. H.; Lee J. H.; Choi H. S.; Yano H.; Zhuo L.; Kurisawa M.; Ying J. Y. Self-Assembled Micellar Nanocomplexes Comprising Green Tea Catechin Derivatives and Protein Drugs for Cancer Therapy. Nat. Nanotechnol 2014, 9 (11), 907–912. 10.1038/nnano.2014.208. 25282044 PMC4221637

[ref20] Lin M.; Paolillo V.; Le D. B.; Macapinlac H.; Ravizzini G. C. Monoclonal Antibody Based Radiopharmaceuticals for Imaging and Therapy. Curr. Probl. Cancer 2021, 45 (5), 100796 10.1016/j.currproblcancer.2021.100796. 34657748

[ref21] Vosjan M. J. W. D.; Perk L. R.; Visser G. W. M.; Budde M.; Jurek P.; Kiefer G. E.; Van Dongen G. A. M. S. Conjugation and Radiolabeling of Monoclonal Antibodies with Zirconium-89 for PET Imaging Using the Bifunctional Chelate p-Isothiocyanatobenzyl-Desferrioxamine. Nat. Protoc. 2010, 5 (4), 739–743. 10.1038/nprot.2010.13. 20360768

[ref22] Pang J.; Xing H.; Sun Y.; Feng S.; Wang S. Non-Small Cell Lung Cancer Combination Therapy: Hyaluronic Acid Modified, Epidermal Growth Factor Receptor Targeted, PH Sensitive Lipid-Polymer Hybrid Nanoparticles for the Delivery of Erlotinib plus Bevacizumab. Biomed. Pharmacother. 2020, 125, 109861 10.1016/j.biopha.2020.109861. 32070872

[ref23] Baião A.; Sousa F.; Oliveira A. V.; Oliveira C.; Sarmento B. Effective Intracellular Delivery of Bevacizumab via PEGylated Polymeric Nanoparticles Targeting the CD44v6 Receptor in Colon Cancer Cells. Biomater. Sci. 2020, 8 (13), 3720–3729. 10.1039/D0BM00556H. 32500879

[ref24] Zhang N.; Song J.; Liu Y.; Liu M.; Zhang L.; Sheng D.; Deng L.; Yi H.; Wu M.; Zheng Y.; Wang Z.; Yang Z. Photothermal Therapy Mediated by Phase-Transformation Nanoparticles Facilitates Delivery of Anti-PD1 Antibody and Synergizes with Antitumor Immunotherapy for Melanoma. J. Controlled Release 2019, 306, 15–28. 10.1016/j.jconrel.2019.05.036. 31132380

[ref25] Henriksen J. R.; Petersen A. L.; Hansen A. E.; Frankær C. G.; Harris P.; Elema D. R.; Kristensen A. T.; Kjær A.; Andresen T. L. Remote Loading of 64Cu2+ into Liposomes without the Use of Ion Transport Enhancers. ACS Appl. Mater. Interfaces 2015, 7 (41), 22796–22806. 10.1021/acsami.5b04612. 26426093

[ref26] Hansen A. E.; Petersen A. L.; Henriksen J. R.; Boerresen B.; Rasmussen P.; Elema D. R.; Rosenschöld P. M. A.; Kristensen A. T.; Kjær A.; Andresen T. L. Positron Emission Tomography Based Elucidation of the Enhanced Permeability and Retention Effect in Dogs with Cancer Using Copper-64 Liposomes. ACS Nano 2015, 9 (7), 6985–6995. 10.1021/acsnano.5b01324. 26022907

[ref27] Le Basle Y.; Chennell P.; Tokhadze N.; Astier A.; Sautou V. Physicochemical Stability of Monoclonal Antibodies: A Review. J. Pharm. Sci. 2020, 109 (1), 169–190. 10.1016/j.xphs.2019.08.009. 31465737

[ref28] Yoon J. T.; Longtine M. S.; Marquez-Nostra B. V.; Wahl R. L. Evaluation of Next-Generation Anti-CD20 Antibodies Labeled with 89ZR in Human Lymphoma Xenografts. J. Nucl. Med. 2018, 59 (8), 1219–1224. 10.2967/jnumed.117.203299. 29348316 PMC6071500

[ref29] Hu G.; Zhu W.; Liu Y.; Wang Y.; Zhang Z.; Zhu S.; Duan W.; Zhou P.; Fu C.; Li F.; Huo L. Development and Comparison of Three 89Zr-Labeled Anti-CLDN18.2 Antibodies to Noninvasively Evaluate CLDN18.2 Expression in Gastric Cancer: A Preclinical Study. Eur. J. Nucl. Med. Mol. Imaging 2022, 49 (8), 2634–2644. 10.1007/s00259-022-05739-3. 35347439

[ref30] Ramos-Membrive R.; Erhard Á.; Luis de Redín I.; Quincoces G.; Collantes M.; Ecay M.; Irache J. M.; Peñuelas I. In Vivo SPECT-CT Imaging and Characterization of Technetium-99m-Labeled Bevacizumab-Loaded Human Serum Albumin Pegylated Nanoparticles. J. Drug Delivery Sci. Technol. 2021, 64, 101809 10.1016/j.jddst.2020.101809.

[ref31] Nikpoor A. R.; Tavakkol-Afshari J.; Sadri K.; Jalali S. A.; Jaafari M. R. Improved Tumor Accumulation and Therapeutic Efficacy of CTLA-4-Blocking Antibody Using Liposome-Encapsulated Antibody: In Vitro and in Vivo Studies. Nanomedicine 2017, 13 (8), 2671–2682. 10.1016/j.nano.2017.08.010. 28847682

[ref32] Behzadi S.; Serpooshan V.; Sakhtianchi R.; Müller B.; Landfester K.; Crespy D.; Mahmoudi M. Protein Corona Change the Drug Release Profile of Nanocarriers: The “Overlooked” Factor at the Nanobio Interface. Colloids Surf., B 2014, 123, 143–149. 10.1016/j.colsurfb.2014.09.009. 25262409

[ref33] Bhatt N. B.; Pandya D. N.; Rideout-Danner S.; Gage H. D.; Marini F. C.; Wadas T. J. A Comprehensively Revised Strategy That Improves the Specific Activity and Long-Term Stability of Clinically Relevant 89 Zr-Immuno-PET Agents. Dalton Trans. 2018, 47 (37), 13214–13221. 10.1039/C8DT01841C. 30178793 PMC6192516

[ref34] Gawne P. J.; Man F.; Blower P. J.; De Rosales R. T. M. Direct Cell Radiolabeling for in Vivo Cell Tracking with PET and SPECT Imaging. Chem. Rev. 2021, 122, 10266–10318. 10.1021/acs.chemrev.1c00767. PMC9185691 35549242

[ref35] Xie X.; Yu T.; Li X.; Zhang N.; Foster L. J.; Peng C.; Huang W.; He G. Recent Advances in Targeting the “Undruggable” Proteins: From Drug Discovery to Clinical Trials. Signal Transduction Targeted Ther. 2023, 8 (1), 335 10.1038/s41392-023-01589-z. PMC10480221 37669923

[ref36] Tang Y.; Pu X.; Yuan X.; Pang Z.; Li F.; Wang X. Targeting KRASG12D Mutation in Non-Small Cell Lung Cancer: Molecular Mechanisms and Therapeutic Potential. Cancer Gene Ther. 2024, 31 (7), 961–969. 10.1038/s41417-024-00778-4. 38734764 PMC11257988

[ref37] Wu F.; Tamhane M.; Morris M. E. Pharmacokinetics, Lymph Node Uptake, and Mechanistic PK Model of near-Infrared Dye-Labeled Bevacizumab after IV and SC Administration in Mice. AAPS J. 2012, 14 (2), 252–261. 10.1208/s12248-012-9342-9. 22391791 PMC3326166

[ref38] Dos Santos N.; Allen C.; Doppen A. M.; Anantha M.; Cox K. A. K.; Gallagher R. C.; Karlsson G.; Edwards K.; Kenner G.; Samuels L.; Webb M. S.; Bally M. B. Influence of Poly(Ethylene Glycol) Grafting Density and Polymer Length on Liposomes: Relating Plasma Circulation Lifetimes to Protein Binding. Biochim. Biophys. Acta 2007, 1768 (6), 1367–1377. 10.1016/j.bbamem.2006.12.013. 17400180

[ref39] Mui B. L.; Tam Y. K.; Jayaraman M.; Ansell S. M.; Du X.; Tam Y. Y. C.; Lin P. J. C.; Chen S.; Narayanannair J. K.; Rajeev K. G.; Manoharan M.; Akinc A.; Maier M. A.; Cullis P.; Madden T. D.; Hope M. J. Influence of Polyethylene Glycol Lipid Desorption Rates on Pharmacokinetics and Pharmacodynamics of SiRNA Lipid Nanoparticles. Mol. Ther. Nucleic Acids 2013, 2, e139 10.1038/mtna.2013.66. 24345865 PMC3894582

[ref40] Miedema I. H. C.; Zwezerijnen G. J. C.; Huisman M. C.; Doeleman E.; Mathijssen R. H. J.; Lammers T.; Hu Q.; van Dongen G. A. M. S.; Rijcken C. J. F.; Vugts D. J.; van Oordt C. W. PET-CT Imaging of Polymeric Nanoparticle Tumor Accumulation in Patients. Adv. Mater. 2022, 34 (21), 2201043 10.1002/adma.202201043. 35427430

[ref41] Yu Y.; Huang C.; Chen F.; Pan W.; Zhang L. Hyaluronan-Based Theranostic Nanomicelles for Breast Cancer-Targeting and Anticancer Drug Delivery. Mater. Des. 2023, 225, 111551 10.1016/j.matdes.2022.111551.

[ref42] He P.; Tang H.; Zheng Y.; Xiong Y.; Cheng H.; Li J.; Zhang Y.; Liu G. Advances in Nanomedicines for Lymphatic Imaging and Therapy. J. Nanobiotechnol. 2023, 21 (1), 292 10.1186/s12951-023-02022-x. PMC10463797 37620846

[ref43] Schudel A.; Francis D. M.; Thomas S. N. Material Design for Lymph Node Drug Delivery. Nat. Rev. Mater. 2019, 4 (6), 415–428. 10.1038/s41578-019-0110-7. 32523780 PMC7286627

[ref44] Raavé R.; Sandker G.; Adumeau P.; Jacobsen C. B.; Mangin F.; Meyer M.; Moreau M.; Bernhard C.; Da Costa L.; Dubois A.; Goncalves V.; Gustafsson M.; Rijpkema M.; Boerman O.; Chambron J. C.; Heskamp S.; Denat F. Direct Comparison of the in Vitro and in Vivo Stability of DFO, DFO* and DFOcyclo* for 89Zr-ImmunoPET. Eur. J. Nucl. Med. Mol. Imaging 2019, 46 (9), 1966–1977. 10.1007/s00259-019-04343-2. 31161258 PMC6647232

