# Supplementary material for: Biodistribution of 89Zr-Radiolabeled Nanoassemblies for Monoclonal Antibody Delivery Revealed through In Vivo PET Imaging
Source: ACS Omega. 2025 Jan 28;10(5):4763–73. doi: 10.1021/acsomega.4c09823 (PMC11822718; doi:10.1021/acsomega.4c09823)
Supplement: Supplementary file 1 — ao4c09823_si_001.pdf [file ao4c09823_si_001.pdf]

SUPPLEMENTARY INFORMATION

Biodistribution of ^{89}Zr-radiolabelled nanoassemblies for monoclonal antibody delivery revealed through *in vivo* PET imaging

Ana M. López-Estévez[1,2], Amaia Carrascal-Miniño[3], Dolores Torres[2], María José Alonso[1,2], Rafael T. M. de Rosales[3*] and Juan Pellico[3*]

[1]Center for Research in Molecular Medicine and Chronic Diseases (CiMUS), Health Research Institute of Santiago de Compostela, University of Santiago de Compostela, 15782, Santiago de Compostela, Spain

[2]Department of Pharmacology, Pharmacy and Pharmaceutical Technology, School of Pharmacy, University of Santiago de Compostela, 15782, Santiago de Compostela, Spain

[3]School of Biomedical Engineering & Imaging Sciences, King's College London, St. Thomas' Hospital, London SE1 7EH, UK.

Corresponding authors: Rafael T. M. de Rosales (rafael.torres@kcl.ac.uk) and Juan Pellico (jpellico@icmab.es)

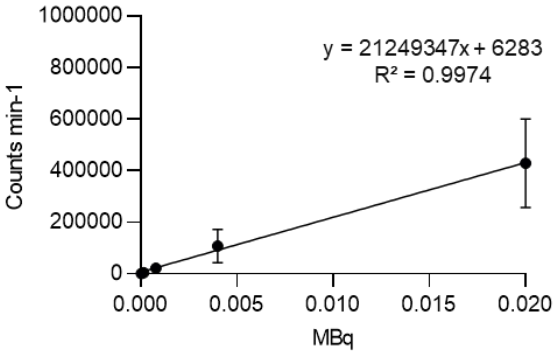

Supplementary Figure 1. Calibration curve of ^{89}Zr for the correlation of MBq and counts per minute. The linear regression was calculated only with the mean value of each point. Data are represented as mean ± SD (n=3).

*Dr Juan Pellico current address: The Institute of Materials Science of Barcelona (ICMAB-CSIC)
Campus de la UAB, 08193, Bellaterra (Barcelona, Spain)

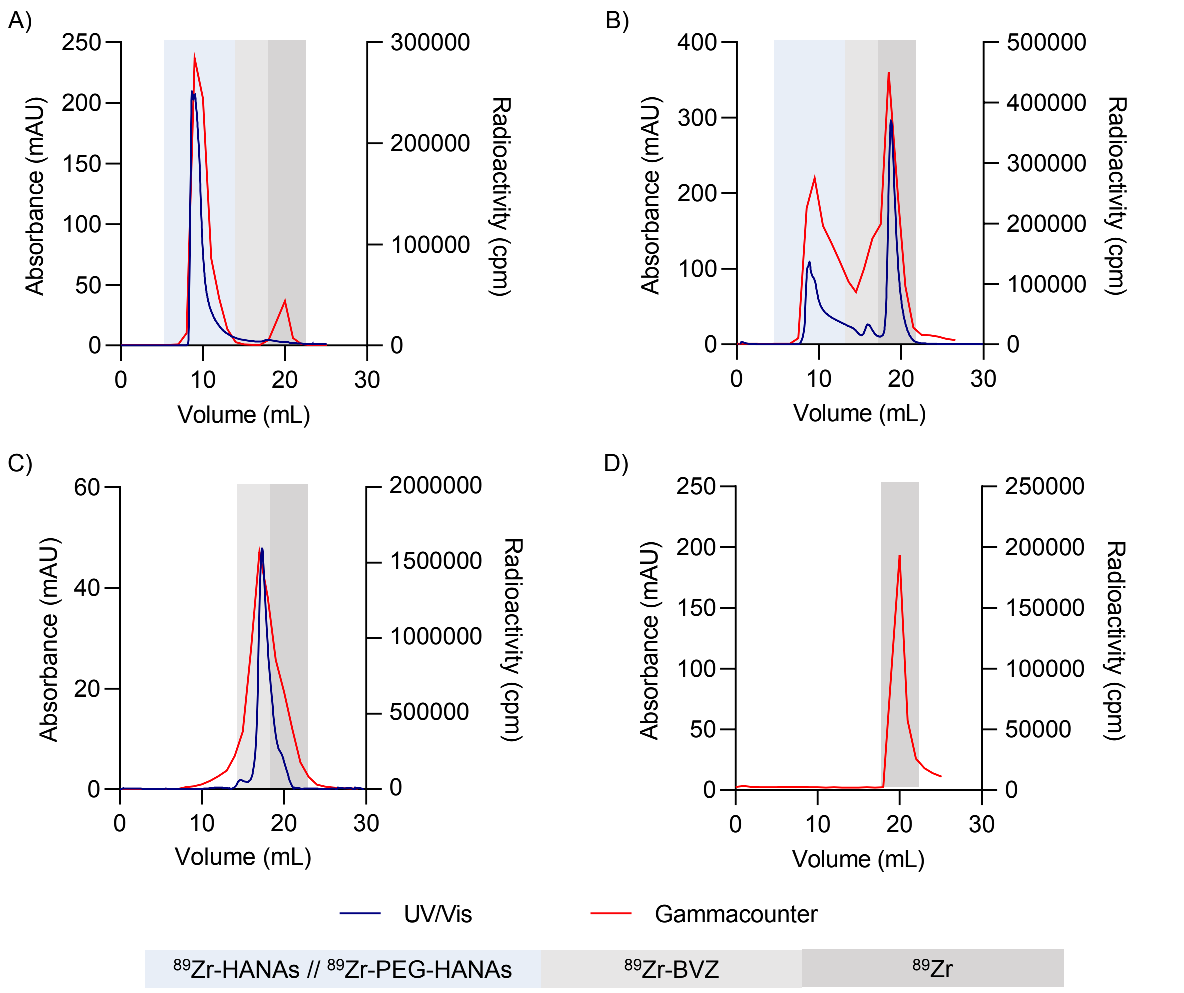

Supplementary Figure 2. UV/Vis and radioactivity profile of HANAs (A) and PEG-HANAs (B) during the ^{89}Zr-labeling and post-purification procedure. Graphs indicate the process required for BVZ removal. As control, the radioactivity profile of ^{89}Zr-BVZ (C) and free ^{89}Zr (D) were analysed. Fractions eluted in the following order based on their size: ^{89}Zr-HANAs or ^{89}Zr-PEG-HANAs, ^{89}Zr-BVZ and ^{89}Zr are indicated. UV absorbance at 280 nm was obtained after using the ÄKTA system and counts per minute (cpm) after γ-counted the eluted fractions. mAU milli-absorption units.

$^{89}ZrCl_4$

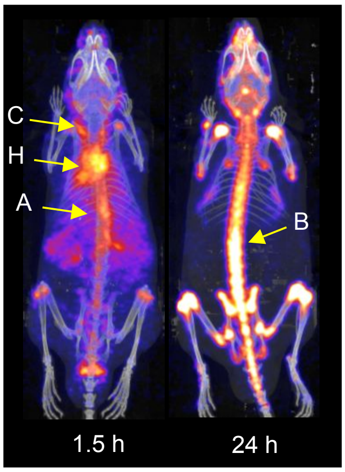

Supplementary Figure 3. PET/CT images of $^{89}Zr\text{-}Cl_4$ ($^{89}Zr\text{-}Cl_4$) in 9 weeks old healthy C57BL/6J mice. PET/CT maximum intensity projections of 1-2 MBq of $^{89}Zr\text{-}Cl_4$ at 1.5 and 24 after i.v. administration (n=4). A aorta, B bone, C carotids, H heart
